# Multiparametric magnetic resonance imaging-based radiomics nomogram for predicting tumor grade in endometrial cancer

**DOI:** 10.3389/fonc.2023.1081134

**Published:** 2023-02-21

**Authors:** Xiaoning Yue, Xiaoyu He, Shuaijie He, Jingjing Wu, Wei Fan, Haijun Zhang, Chengwei Wang

**Affiliations:** ^1^ Department of CT&MRI, The First Affiliated Hospital of Medical College, Shihezi University, Shihezi, China; ^2^ Department of Pathology, The First Affiliated Hospital of Medical College, Shihezi University, Shihezi, China

**Keywords:** endometrial cancer, histological grade, magnetic resonance imaging, radiomics, nomogram

## Abstract

**Background:**

Tumor grade is associated with the treatment and prognosis of endometrial cancer (EC). The accurate preoperative prediction of the tumor grade is essential for EC risk stratification. Herein, we aimed to assess the performance of a multiparametric magnetic resonance imaging (MRI)-based radiomics nomogram for predicting high-grade EC.

**Methods:**

One hundred and forty-three patients with EC who had undergone preoperative pelvic MRI were retrospectively enrolled and divided into a training set (*n* =100) and a validation set (*n* =43). Radiomic features were extracted based on T2-weighted, diffusion-weighted, and dynamic contrast-enhanced T1-weighted images. The minimum absolute contraction selection operator (LASSO) was implemented to obtain optimal radiomics features and build the rad-score. Multivariate logistic regression analysis was used to determine the clinical MRI features and build a clinical model. We developed a radiomics nomogram by combining important clinical MRI features and rad-score. A receiver operating characteristic (ROC) curve was used to evaluate the performance of the three models. The clinical net benefit of the nomogram was assessed using decision curve analysis (DCA), net reclassification index (NRI), and integrated discrimination index (IDI).

**Results:**

In total, 35/143 patients had high-grade EC and 108 had low-grade EC. The areas under the ROC curves of the clinical model, rad-score, and radiomics nomogram were 0.837 (95% confidence interval [CI]: 0.754–0.920), 0.875 (95% CI: 0.797–0.952), and 0.923 (95% CI: 0.869–0.977) for the training set; 0.857 (95% CI: 0.741–0.973), 0.785 (95% CI: 0.592–0.979), and 0.914 (95% CI: 0.827–0.996) for the validation set, respectively. The radiomics nomogram showed a good net benefit according to the DCA. NRIs were 0.637 (0.214–1.061) and 0.657 (0.079–1.394), and IDIs were 0.115 (0.077–0.306) and 0.053 (0.027–0.357) in the training set and validation set, respectively.

**Conclusion:**

The radiomics nomogram based on multiparametric MRI can predict the tumor grade of EC before surgery and yield a higher performance than that of dilation and curettage.

## Introduction

The incidence of endometrial carcinoma (EC) has risen steadily in recent years and the standard operation for EC consists of hysterectomy and bilateral salpingo-oophorectomy (1, 2). The 2020 the European Society of Gynaecological Oncology the European Society for Radiotherapy & Oncology and the European Society of Pathology (ESGO-ESTRO-ESP) guidelines recommend pelvic and abdominal para-aortic lymph node dissection for patients with high-intermediate-risk/high-risk EC (high-grade EC and myometrial invasion ≥ 50%), but not low-risk EC (low-grade EC, myometrial invasion< 50%, and lymphatic vascular space invasion [LVSI] negative) (3). The prognosis of EC is related to tumor grade, deep myometrial invasion (DMI), LVSI, and lymph node metastasis (LNM). Tumor grade is an important predictor of disease outcome and LNM as well as an important cornerstone for determining the extent of surgical treatment (4, 5).

Almost all patients with EC undergo preoperative dilation and curettage (D&C) or hysteroscopic biopsy. A recent review showed moderate agreement between D&C and the final surgical pathology (6). The underestimation of the pathological grade will lead to inadequate treatment and risk of LNM in the future, whereas overestimation of the pathological grade will lead to excessive surgical treatment and cause unnecessary complications in patients (7, 8). One study showed that the inconsistent diagnosis of preoperative pathological grading is an important reason for the high mortality rate (9). Consequently, it is necessary to develop an accurate and noninvasive preoperative method to predict the tumor grade of EC.

In addition to diagnostic curettage, magnetic resonance imaging (MRI) has the greatest potential to predict tumor grade. Most studies have predicted the pathological grade of EC using conventional MRI features or apparent diffusion coefficient (ADC) values (10, 11). However, owing to the subjective influence of measurement level and experience, some quantitative indicators are difficult to represent the heterogeneity of the whole tumor. Their value in evaluating tumor grade remains controversial. Radiomics is a non-invasive method for quantitatively assessing tumor heterogeneity by digitally analyzing a large number of image features extracted from medical images with high throughput. In addition, radiomics can link image features with phenotypes by establishing descriptive and predictive models, which may provide useful information for differential tumor diagnosis and evaluation of tumor response to treatment (12–14). In EC, previous studies have demonstrated that radiomics performs well in assessing the depth of myometrial invasion (MI), LVSI, LNM, and prognosis (9, 15–17). Therefore, we believe that radiomics is a promising tool for predicting preoperative tumor grade.

This study aimed to develop a radiomics nomogram based on multiparametric MRI to predict high-grade EC and compare the net clinical benefit of the radiomics nomogram with that of preoperative D&C.

## Materials and methods

### Patients

**Figure 1:**
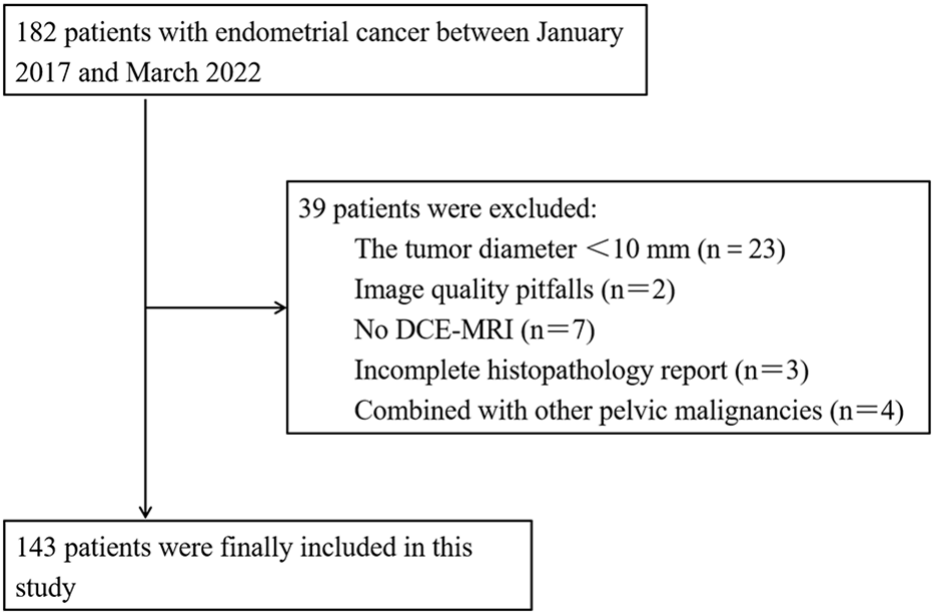
Flowchart of included and excluded patients with endometrial cancer.

This study was approved by the Ethics Committee of our institution and the requirement for patient informed consent was waived. Between January 2017 and March 2022, 182 patients with a histopathological diagnosis of EC underwent preoperative pelvic MRI. The inclusion criteria were as follows: (1) patients with EC confirmed by postoperative histopathology. (2) MRI was performed within 2 weeks before the operation in our hospital, and (3) no adjuvant therapy was performed before MRI examination. The exclusion criteria were as follows: (1) tumor was less than two layers on MRI or the maximum diameter of the tumor was less than 10 mm (*n* = 23), (2) image quality pitfalls (*n* = 2), (3) no DCE-MRI (*n* = 7), (4) incomplete histopathology report (*n* = 3), and (5) combined with other pelvic malignancies (*n* = 4). Finally, a total of 143 patients (average age 55.52 ± 10.46 years) were enrolled and randomly divided into the training set (100 patients, 27 of whom had high-grade EC) and the validation set (43 patients, eight of whom had high-grade EC) at a ratio of 7:3 by stepwise sampling. A flow chart of the inclusion and exclusion criteria for the patients is shown in **Figure 1**.

### MRI protocols

Axial TI-weighted imaging (T1WI), sagittal and coronal T2-weighted imaging (T2WI) without fat suppression, axial fat suppression T2WI, axial diffusion-weighted imaging (DWI [*b* = 0 and 800 s/mm^2^]), and three planes (axial, sagittal, and coronal) of dynamic contrast-enhanced T1-weighted images (DCE-T1WI) of the pelvis were performed using a 3.0 T magnetic resonance machine (GE Discovery MR 750 W, Milwaukee, WI) and one 1.5 T MR machine (Philips, Maltiva, the Netherlands). All the images were acquired using an eight-channel phased array surface coil. The patients fasted for 4–6h before MRI scans to reduce artifacts caused by bowel peristalsis. There were eight dynamic phases in DCE-T1WI. The first was a mask film. Before the second dynamic phase scanning, a contrast agent (gadolinium chelate, GE Healthcare) was injected into the cubital vein of the patient with a dosage of 0.2 ml/kg and an injection rate of 2–3 ml/s. Each dynamic phase was scanned for 18–20 s. The details of the MRI scanning protocols are listed in **Supplementary Table S1A**.

### Classification of tumor grade

Two pathologists divided endometrioid adenocarcinomas into well differentiated (grade 1), moderately differentiated (grade 2), and poorly differentiated (grade 3) according to the proportion of non-squamous solid components in the tumor tissue (18). For the difference in 5-year survival and prognosis, we considered grade 1/grade 2 endometrioid adenocarcinoma as low-grade EC, grade 3 adenocarcinoma, and non-endometrioid adenocarcinoma (e.g., clear, serous cell carcinomas, etc.) as high-grade EC, which has a less favorable prognosis (1).

### Clinical and conventional MRI features

Clinical data, including patient age, CA125 (within 2 weeks before surgery), HE4 (within 2 weeks before surgery), and tumor grade by preoperative D&C, were obtained through the hospital information management system. Pathological reports should include tumor differentiation, depth of MI, CSI, and FIGO stage.

Two radiologists (A and B with 5 and 12 years of experience, respectively) reviewed the MRI images of each patient, blinded to the pathological and clinical data. The evaluation items included maximum tumor diameter (mean value of the tumor on axial T2WI, DWI, and DCE-T1WI), depth of MI, CSI, and LNM. Disagreements were re-evaluated by another senior physician.

### Image segmentation and radiomics feature extraction

**Figure 2:**
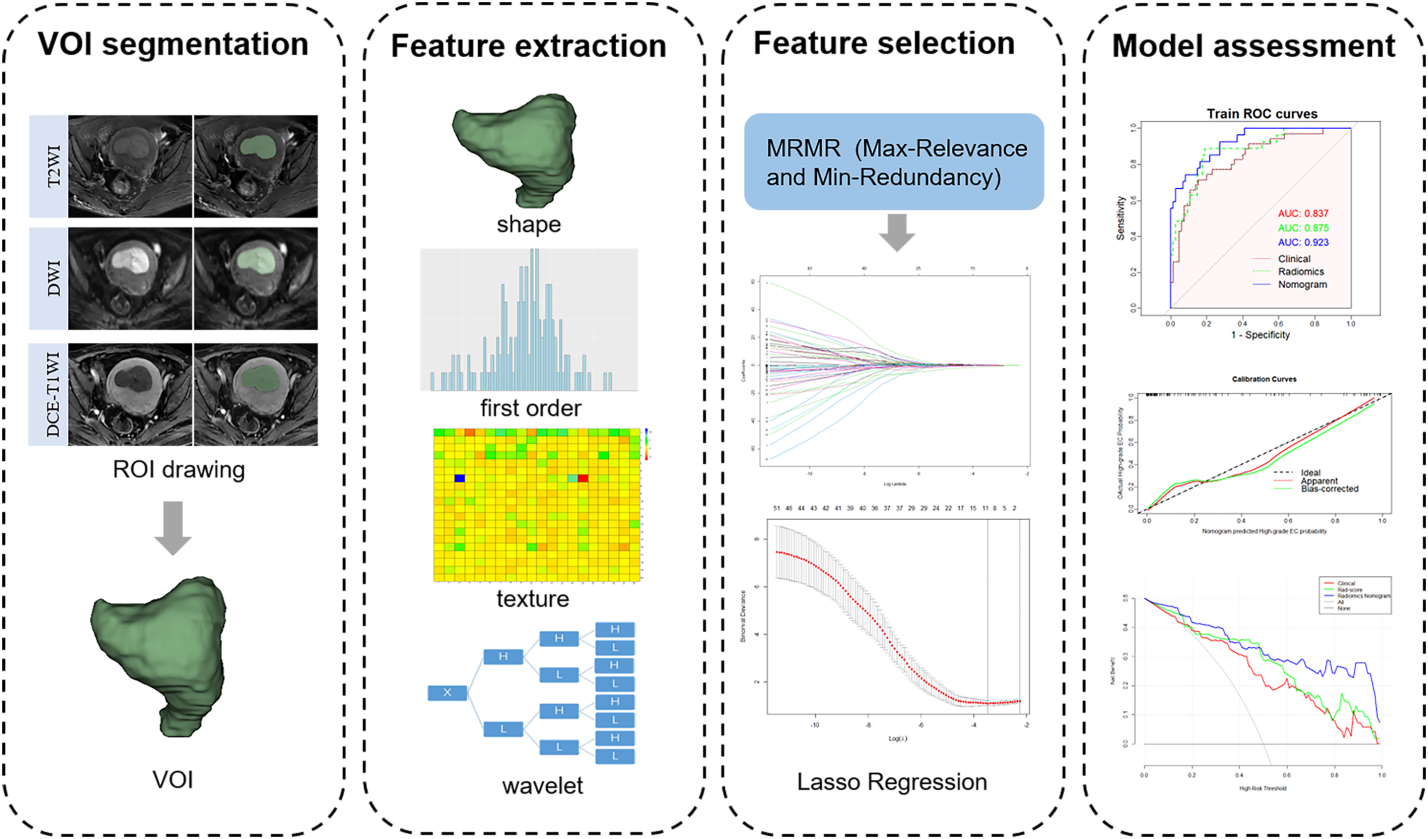
Workflow of radiomics analysis and model building.

The region of interest (ROI) was manually delineated in each layer of the tumor on axial T2WI, DWI, and DCE-T1WI images (the seventh dynamic scanning period) by radiologist A and automatically converted into three-dimensional images to obtain the volume of interest (VOI) using the 3D-Slicer software (v.4.11.0, https://www.slicer.org). Subsequently, radiologist B randomly selected 40 patients to draw the ROI in the same manner. All ROIs were drawn considering cystic, necrotic, and bleeding areas within the tumor, but avoiding the normal muscularis adjacent to the tumor tissue and hematoma outside the tumor. A flowchart of the radiomics feature extraction is shown in **Figure 2**.

Before extracting radiomics features, MRI images must be pre-processed to compensate for the difference in signal intensity caused by different field strengths and scanning protocols. Image preprocessing included resampling the image to a voxel size of 1 mm^3^ and discretizing the voxel intensity value with a fixed bin width of 25 mm to standardize the gray intensity of each image and reduce image noise.

In total, 851 radiomics features extracted from each VOI of T2WI, DWI, and DCE-T1WI images included shape-based, first-order, and texture features (including GLCM, GLDM, GLSZM, GLRLM, and NGTDM). The intraclass correlation coefficient (ICC) was used to evaluate the reproducibility of radiomics features. To explore more information inside the tumor to highlight the differences between tumor grades, the first-order features and texture features were transformed by wavelet transform, and eight wavelet decomposition features of different frequency bands were obtained. Detailed information on all the features is provided in **Supplementary Table S2A**. All radiomics features were preprocessed using *Z*-score standardization to eliminate the influence of different gray values.

### Features selection and radiomics score construction

The radiomics features with ICC ≥ 0.75 into R software (v4.2.0, https://www.R-project.org). First, 80 radiomics features with the greatest correlation with tumor grade were selected based on the maximum relevance and minimum redundancy (mRMR) algorithm. These features were further reduced in dimension and screened using least absolute shrinkage and selection operator (LASSO) regression. The regularization parameter λ was adjusted by 10-fold cross-validation to select robust features and construct a radiomics score (rad-score) by linear combinations weighted by the corresponding coefficients of the selected features.

### Development of clinical model and radiomics nomogram

Univariate and multivariate logistic regression analyses were used for clinical and conventional MRI features associated with tumor grade. Features with statistically significant differences were considered independent risk factors and were used to establish the clinical model. Next, a radiomics nomogram was established by combining the above independent risk factors with the rad-score using logistic regression. A calibration curve was drawn, and the *p*-value of the Hosmer-Lemeshow test was used to evaluate the fitting effect of the model.

### Clinical usefulness

The clinical feasibility of the radiomics nomogram, rad-score and clinical model was evaluated by decision curve analysis (DCA). The net benefits of both under different probability thresholds were analyzed by comparing the clinical decision curves of the radiomics nomogram and preoperative D&C. The net reclassification index (NRI) and integrated discrimination index (IDI) were calculated to analyze the advantages of the radiomics nomogram in predicting high-grade EC compared with those of D&C. Finally, the clinical impact curve (CIC) was used to analyze the loss-benefit ratio of the nomogram and preoperative D&C compared with the actual postoperative pathological results of each patient under different probability thresholds.

### Statistical analysis

The normality of all parameters was checked using the Shapiro–Wilk test. Quantitative data were analyzed using the *t*-test or Mann-Whitney *U* test, and qualitative data were analyzed using the chi-square test. Stepwise logistic regression was performed to establish models for predicting high-grade EC from the statistically significant variables. The predictive performance indicators obtained in the training and validation sets included receiver operating characteristic (ROC) curves and correlation areas under the curve (AUCs). The prediction efficiency of the models was compared using the Delong’ test. *P*< 0.05 indicates statistical significance. Statistical analysis of all data was conducted using the R software (v4.2.0, https://www.R-project.org). The “Irr” package was used for ICC analysis. The “mRMRe” package and “glmnet” package were used for screening and dimensionality reduction of image features. The “rms” package was required to obtain nomogram and calibration curve. The analysis of DCA required the installation of “rmda” package. Finally, NRI and IDI were calculated using “predidicABEL” package.

## Results

### Clinical features and model construction

The clinical and pathological features of the 143 patients were balanced between the training and validation sets, and the difference between the two sets was not statistically significant (**Table 1**). The pathological grade was high-grade EC in of 35/143 patients (24.5%) and low-grade EC in 108/143 patients (75.5%). Univariate *t*-test analysis showed that age, HE4, DMI (MR_DMI), CSI (MR_CSI), and LNM (MR_LNM) on MRI reports were significantly different between high-grade and low-grade ECs, but no statistically significant association between maximum tumor diameter and CA125 and tumor grade was found (**Supplementary Table S3A**). Univariate and multivariate logistic regression analyses indicated that age, MR_DMI, MR_CSI, and MR_LNM were independent risk factors for high-grade EC.

**Table 1:** Patient characteristics.

Characteristics	Training set (n100)	Validation set (n43)	*P* value
Age (y)	55.5±10.5	55.5±10.4	0.979
CA125	45.8±57.4	31.5±31.7	0.128
HE4	107.6±94.8	109.1±90.4	0.933
Tumor size	50.4±20.0	53.5±35.9	0.516
MR_DMI			0.692
Absent	64 (64.0%)	29 (67.4%)	
Present	36 (36.0%)	14 (32.6%)	
MR_CSI			0.435
Absent	78 (78.0%)	36 (83.7%)	
Present	22 (22.0%)	7 (16.3%)	
MR_LNM			0.219
Absent	87 (87.0%)	38 (88.4%)	
Present	13 (13.0%)	5 (11.6%)	
FIGO stage			0.277
IA	48 (48.0%)	27 (62.8%)	
IB	12 (12.0%)	6 (14.0%)	
II	10 (10.0%)	5 (11.6%)	
IIIA	7 (7.0%)	1 (2.3%)	
IIIB	2 (2.0%)	1 (2.3%)	
IIIC	18 (18.0%)	2 (4.7%)	
IVB	3 (3.0%)	1 (2.3%)	
Histopathology DMI			0.202
Absent	66 (66.0%)	33 (76.7%)	
Present	34 (34.0%)	10 (23.3%)	
Histopathology CSI			0.033
Absent	72 (72.0%)	38 (88.4%)	
Present	28 (28.0%)	5 (11.6%)	
Histopathology LNM			0.468
Absent	80 (80.0%)	38 (88.4%)	
Present	20 (20.0%)	5 (11.6%)	

FIGO, Federation of International of Gynecologists and Obstetricians; HE4, human epididymis protein 4; MR_DMI, MRI-reported deep myometrium invasion; MR_CSI, MRI-reported cervical stromal invasion; MR_LNM, MR-reported lymph node metastasis

Discordance between the preoperative D&C and final pathological results was observed in 34/143 patients (23.8%). The tumor grade in 24/143 patients (16.8%) was underestimated, of which 10/24 patients (41.7%) with preoperative grade 1/2 were found to be grade 3, and 14/24 patients (58.3%) with preoperative grade 1 were found to be grade 2. In contrast, the pathological grade was overestimated in 10/143 patients (7.0%), including 3/10 grade 1/grade 2 patients (30.0%) who were preoperatively diagnosed with grade 3 or non-endometrioid cancer and 7/10 grade 1 patients (70.0%) who were preoperatively diagnosed with grade 2.

Two radiologists at our institution retrospectively analyzed the MR images of the patients with EC. The sensitivity for the diagnosis of DMI, CSI, and LNM was 86.4, 66.67, and 40.0%, respectively, and the specificity for the diagnosis of DMI, CSI, and LNM was 89.9, 93.64, and 95.4%, respectively.

### Radiomics features selection and radiomics score development

**Figure 3:**
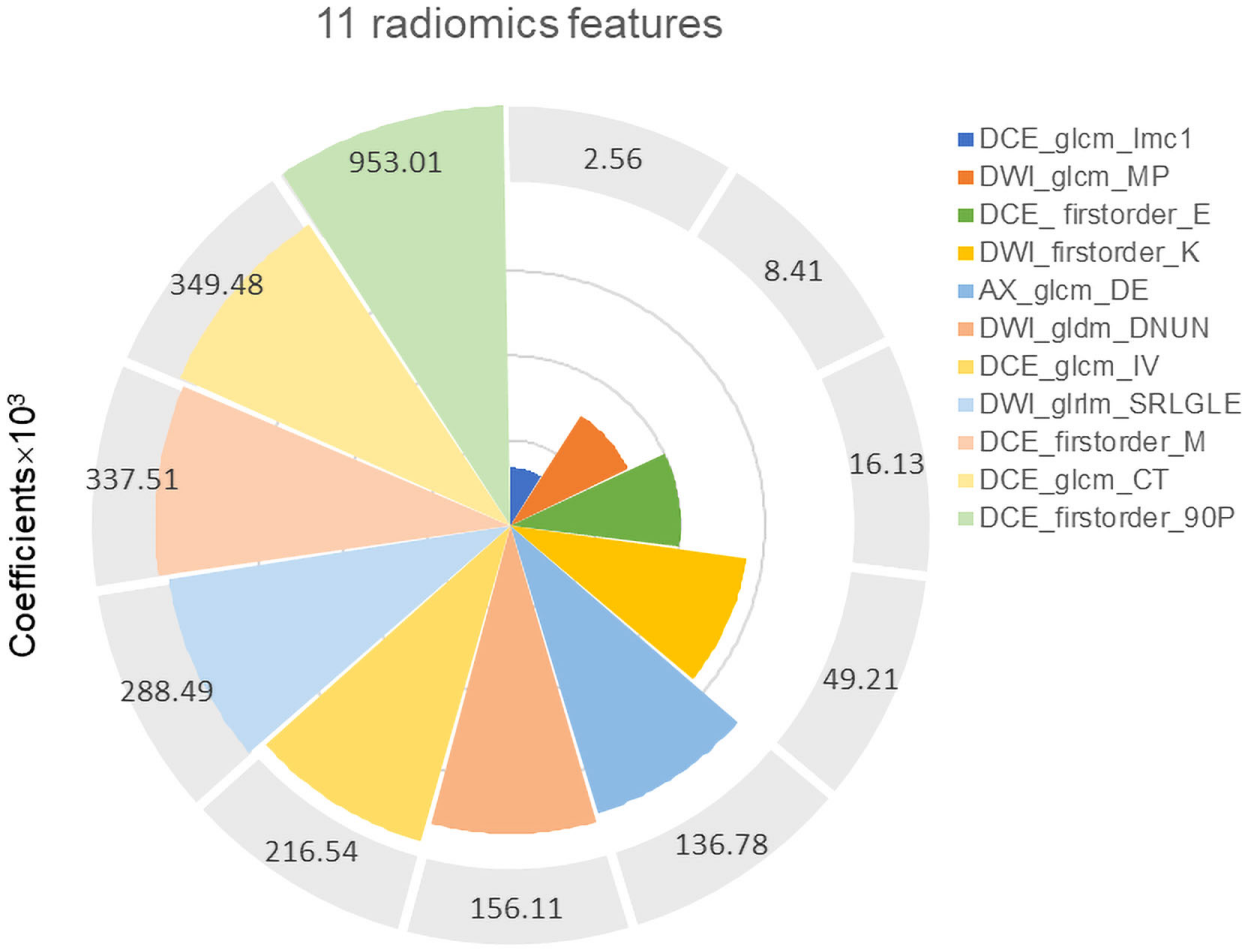
Eleven robust radiomics features and corresponding coefficients for rad-score construction.

After ICC analysis, 2,225 features (739 T2WI features, 736 DWI features, and 750 DCE-T1WI features) were retained. The mRMR algorithm was used to screen out the 80 features most related to high-grade EC, and then LASSO regression was used to avoid radiomics feature overfitting, taking λ as the minimum value (**Supplementary Figure S1A**). Finally, 11 features with nonzero coefficients were retained to construct the rad-score (**Figure 3**). The formula for calculating the rad-score is as follows:


$$\begin{array}{l} Rad-score=-1.1563-0.2885\;*\;M1\;+\;0.0161\;*\;M2-\\ 0.9530\;*\;M3\;+\;0.0026\;*\;M4-0.2165\;*\;M5\\ +\;0.3375\;*\;M6\;+\;0.1561\;*\;M7\;+\;0.1368\;*\;M8\;+\\ \;0.0492\;*\;M9\;+\;0.3495\;*\;M10-0.0084\;*\;M11. \end{array}$$

M1 = DCE_wavelet.LLH_firstorder_Entropy;

M2 = DWI_wavelet.LHH_glrlm_ShortRunLowGrayLevelEmphasis;

M3 = DCE_wavelet.LLH_firstorder_90Percentile;

M4 = DCE_wavelet.HLH_glcm_IMC1;

M5 = DCE_wavelet.LHL_glcm_InverseVariance;

M6 = DCE_original_firstorder_Maximum;

M7 = DWI_wavelet.HHH_gldm_DependenceNonUniformityNormalized;

M8 = AX_wavelet.HHH_glcm_DifferenceEntropy;

M9 = DWI_wavelet.HHH_firstorder_Kurtosis;

M10 = DCE_wavelet.LLH_glcm_ClusterTendency;

M11 = DWI_wavelet.HH_Hglcm_MaximumProbability.

### Prediction performance and validation of radiomics nomogram

**Figure 4:**
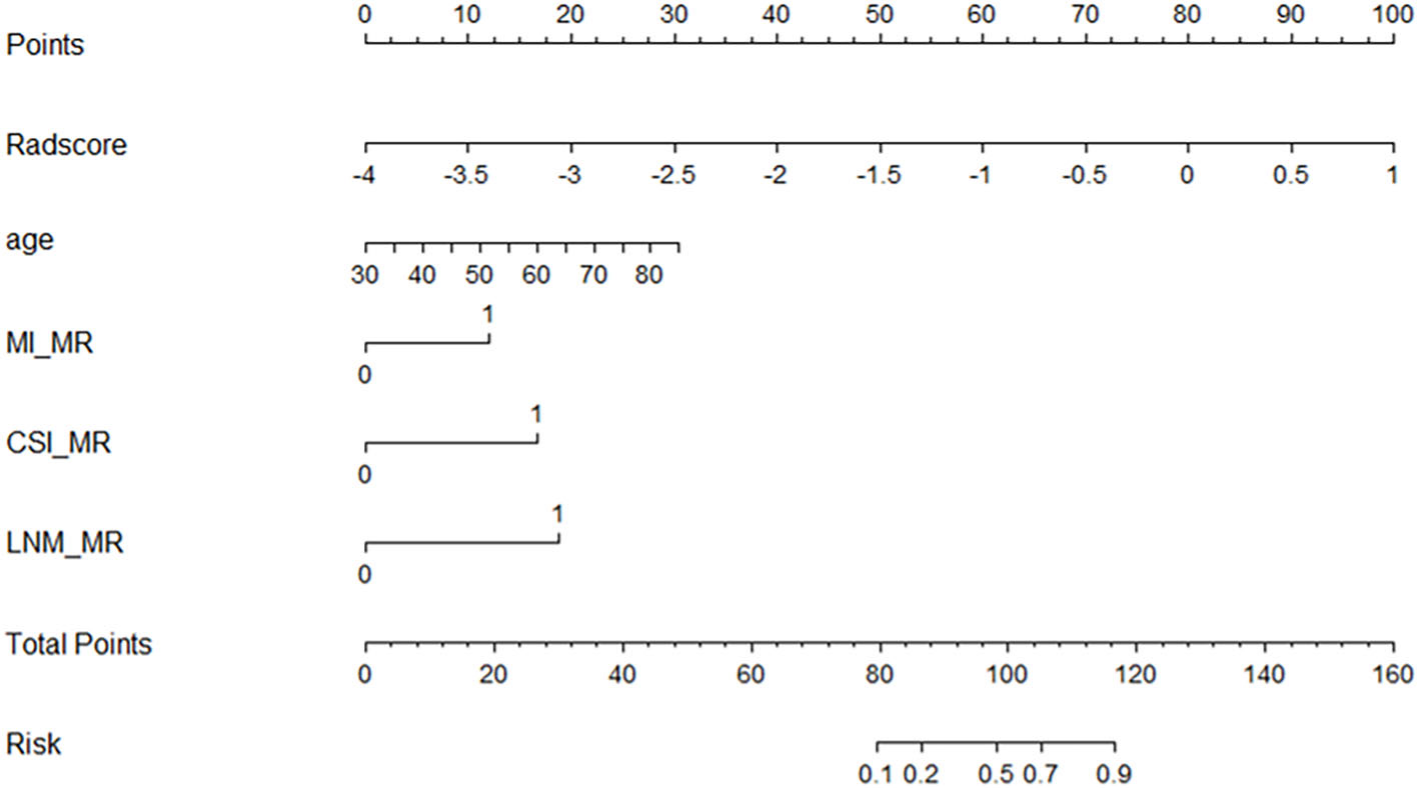
Nomogram for predicting the tumor grade of endometrial cancer, established based on multiparameter magnetic resonance imaging and patient age.

**Figure 5:**
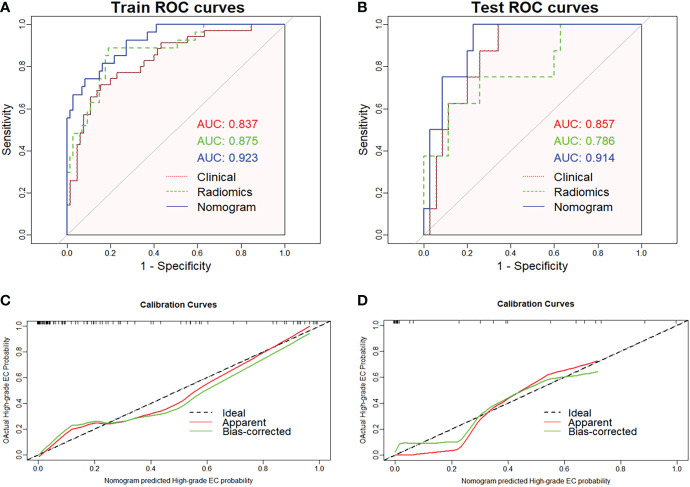
Receiver operating characteristic curves of the three models predicting high-grade endometrial cancer in the training **(A)** and validation sets **(B)**. The graphs **(C)** and **(D)** show that the calibration curve of nomogram has good calibration ability in both the training and validation sets, respectively.

The radiomics nomogram was established using logistic regression by combining the above four clinical and MRI factors (age, MR_DMI, MR_CSI, and MR_LNM) with the rad-score (**Table 2**), which was visualized by the nomogram in **Figure 4**. The AUCs of the clinical model, rad-score, and radiomics nomogram were 0.837 (95% confidence interval [CI]: 0.754–0.920), 0.875 (95% CI: 0.797–0.952) and 0.923 (95% CI: 0.869–0.977) in the training set, and 0.857 (95% CI: 0.741–0.973), 0.786 (95% CI: 0.592–0.979), and 0.914 (95% CI: 0.827–0.998) in the validation set. The prediction performance of the three models is shown in **Table 3**, with the ROC curves shown in **Figures 5A, B**.

**Table 2:** Univariable and multivariable Logistic regression analyses results for high-grade EC.

Characteristics	Univariable analysis		Multivariable analysis	
	OR (95%CI)	*P* value	OR (95%CI)	*P* value
Age	1.084 (1.037, 1.138)	0.001	1.090 (1.012, 1.186)	0.028
HE4	1.049 (1.029, 1.117)	0.014	1.008 (1.001, 1.018)	0.060
MI_MR	6.573 (2.559, 17.874)	0.001	5.268 (1.323, 24.498)	0.023
MR_ CSI	6.500 (2.303, 19.426)	0.001	6.547 (1.287, 39.904)	0.028
MR_LNM	8.625 (2.511, 34.918)	0.001	7.847 (2.106, 35.293)	0.012
Radiomics score	11.031 (4.280, 35.601)	0.001	9.237 (2.723, 43.079)	0.001

OR, odds ratio; CI, confidence interval; EC, endometrial cancer.

**Table 3:** Predictive performance of the clinical model, radiomics score, and radiomics nomogram for high-grade endometrial cancer.

Cohort	Models	AUC (95%CI)	ACC	SEN	SPE	NPV	PPV
Training set	Clinical model	0.837 (0.754, 0.920)	0.801	0.714	0.846	0.846	0.714
	Radiomics score	0.875 (0.797, 0.952)	0.830	0.889	0.808	0.952	0.632
	Radiomics nomogram	0.923 (0.869, 0.977)	0.877	0.741	0.918	0.905	0.769
Validation set	Clinical model	0.857 (0.741, 0.973)	0.721	1.000	0.657	1.000	0.400
	Radiomics score	0.786 (0.592, 0.979)	0.837	0.625	0.886	0.912	0.556
	Radiomics nomogram	0.914 (0.827, 0.998)	0.839	1.000	0.771	1.000	0.500

AUC, area under curve; ACC, accuracy; SEN, sensitivity; SPE, specificity; PPV, positive predictive value, NPV, negative predictive value.

The radiomics nomogram yielded the best prediction performance for both sets. The calibration curves are shown in **Figures 5C, D**, indicating that the nomogram prediction results were in good agreement with the pathological grade of EC in the training and validation sets (*p* = 0.551 and 0.998, respectively). Delong’s test demonstrated that the difference between the nomogram and clinical model was statistically significant in the training and validation sets (*p* = 0.019 and 0.031, respectively). However, the difference between the rad-score and clinical model was not statistically significant (all *p* > 0.05).

### Clinical practicability

**Figure 6:**
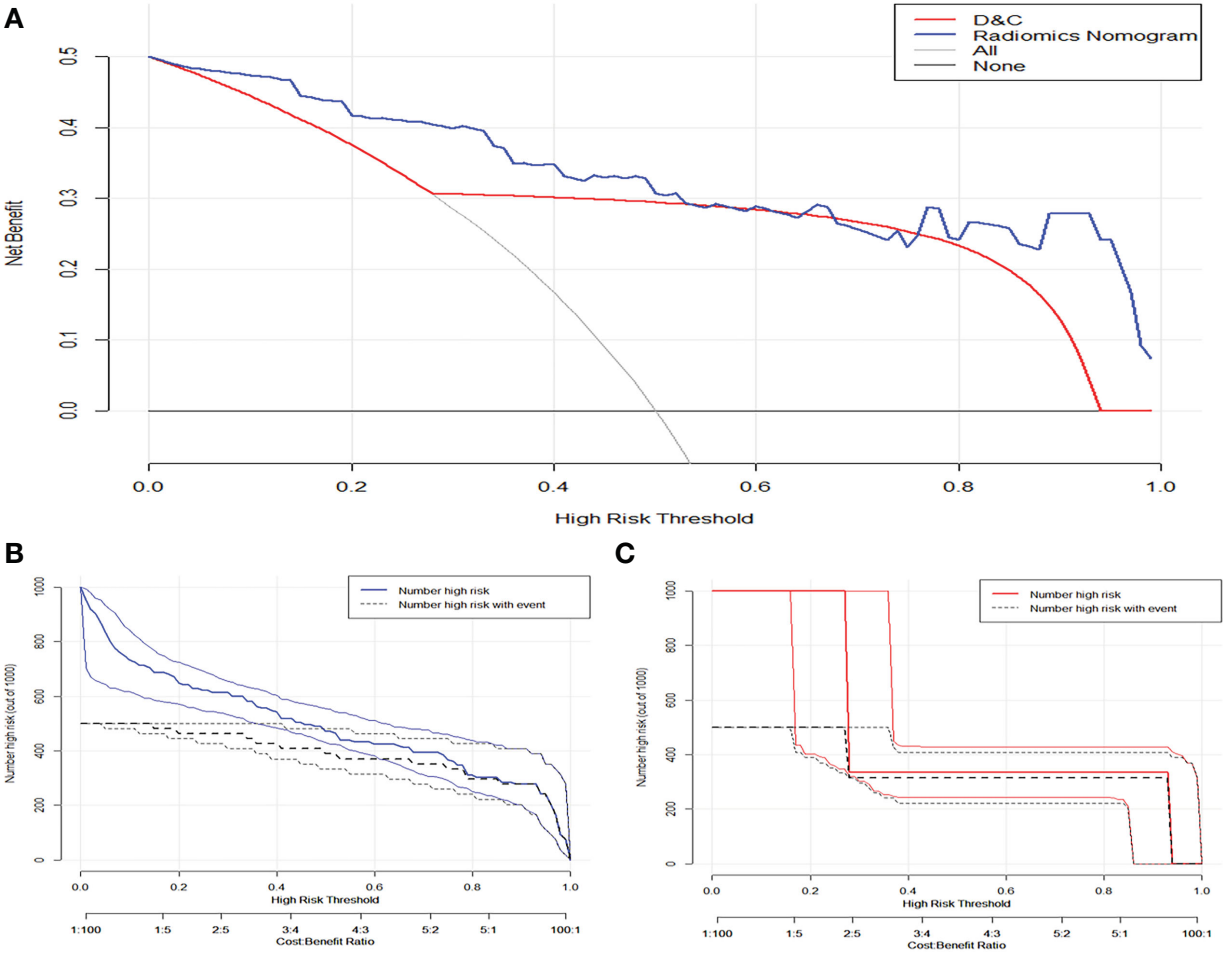
**(A)** The clinical decision curve demonstrated that nomogram has higher net benefits than preoperative curettage at a threshold probability of 0–0.47 and > 0.67. The solid blue and orange lines in figures **(B)** and **(C)** represent the clinical impact curves of the nomogram and the actual preoperative DC, respectively. The black dashed line represents the postoperative pathological results of patients with endometrial cancer, and the closer the solid line is to the black dashed line, the better the prediction effect.

The DCA of the three models showed that the developed radiomics nomogram had a higher net benefit than the rad-score and clinical model at most threshold probabilities in the training (**Figure 6A**) and validation sets (**Supplementary Figure S2A** **),** and a higher net benefit than the actual D&C at threshold probabilities of 0–0.46 and greater than 0.67. The CIC showed the loss-benefit ratio obtained by the radiomics nomogram and D&C at different probability thresholds (**Figures 6B, C**). The reclassification measures of discrimination indicated that, compared with those of D&C, the NRIs of the radiomics nomogram were 0.637 (95% CI: 0.214–1.061, *p* = 0.003) and 0.657 (95% CI: 0.079–1.394, *p* = 0.05), and IDIs of radiomics nomogram were 0.115 (95% CI: 0.077–0.306, *p* = 0.241) and 0.053 (95% CI: 0.027–0.357, *p* = 0.788) in the training set and validation set, respectively.

## Discussion

In this study, we developed a radiomics nomogram based on MRI radiomics features for noninvasive preoperative prediction of tumor grade in EC. The radiomics nomogram can improve the accuracy of distinguishing high-grade EC before surgery, and DCA showed that the nomogram has clinical practicability in assessing preoperative risk stratification of EC. Because the required parameters are easy to obtain, the nomogram is expected to be a powerful tool for gynecologists to develop individualized treatments.

### Predictive value of clinical model for high-grade EC

Two radiologists retrospectively analyzed the MRI images of each patient, and the sensitivity and specificity of the diagnosis of DMI, CSI, and LNM were consistent with those of previous studies (19–21). Many studies (5, 22–24) have confirmed that patient age, DMI, CSI, and LNM are important prognostic factors in high-risk patients with EC. Our study indicated that advanced age, MRI-reported DMI, CSI, and LNM were independent risk factors for high-grade EC. We found that the serum HE4 level of high-grade EC was significantly higher than that of low-grade EC. Although serum HE4 level was not an independent predictor of high-grade EC in this study, serum HE4 was connected with the prognostic factors of tumor grade, FIGO stage, and LNM in EC (25). For gynecologists, preoperative serum HE4 levels are of great clinical value for assessing EC risk stratification. In addition, CA125 was not significantly different between low-grade and high-grade EC, which is inconsistent with the findings of Zheng et al. (26). Serum CA125 is closely related to extrauterine invasion and LNM (20, 27). Therefore, we speculate that this may be caused by different pathological features, such as the FIGO stages. In addition, the mean maximum tumor diameter in the three sequences was not related to tumor grade. Although the clinical model combined conventional MRI features with patient age, ROC and DCA analyses revealed that it had limited usefulness in predicting the pathological grade of EC.

### Predictive value of rad-score for high-grade EC

Radiomics can extract massive features from MRI images, which can effectively solve the problem of tumor heterogeneity that is difficult to quantitatively evaluate (28). In this study, we screened 11 radiomics features that were strongly correlated with tumor grade to construct the rad-score. Among them, the DCE sequence extracted more radiomics features (7/11) than the other two sequences, suggesting that DCE-MRI could provide more tumor information using a contrast agent. The higher the grade of the tumor, the greater the angiogenesis and vascular permeability, which makes the necrotic cystic changes of the tissue more clearly displayed (29). In addition, among all types of radiomics features, high-dimensional abstract wavelet features accounted for the largest proportion, which indicates that wavelet signs can capture clinical information that is not easily perceived visually and can better reflect tumor heterogeneity. Therefore, radiomics can play a significant role in predicting prognostic factors of EC in the future.

### Radiomics nomogram further improved the accuracy of prediction

The radiomics-based nomogram included patient age, MR_DMI, MR_CSI, MR_LNM, and rad-score. Compared with that of the radiomics score and clinical model, the nomogram had improved accuracy, better predictive performance, and higher net benefit. Bereby-Kahane et al. (30) suggested that texture features based on two-dimensional MRI were of limited value in predicting high-grade endometrial adenocarcinoma, with a sensitivity of 52% and a specificity of 75%. A recent study (26) developed a radiomics nomogram based on radiomics features, CA125, and body mass index, with a sensitivity of 88.8% and specificity of 81.5% for predicting high-grade EC. The prediction performance was higher than that of the previous study, but the specificity was lower than that of our study. Unfortunately, only shape features, first-order features, and partial texture features were covered in their study. In our study, the radiomics nomogram not only included conventional MRI features assessed by two radiologists but also feature extraction from multiple sequences (T2WI, DWI, and DCE-MRI), which can provide a practical clinical tool for preoperative risk stratification of EC.

### The nomogram had great potential compared with D&C in predicting tumor grade

Although almost all patients underwent D&C or endometrial biopsy before surgery, the accuracy of preoperative pathological grading evaluation was uneven due to limited tumor tissue samples, tumor heterogeneity, and operator experience. A previous meta-analysis showed a 67% (95% CI: 0.60–0.75) agreement rate between preoperative endometrial sampling and final histopathology, with 21% of tumor grades underestimated and 25% of tumor grades overestimated (31). A recent review (6) obtained similar results and concluded that preoperative EC sampling is not always the best predictor of the final pathological grade of EC. In this study, we found that the concordance rate between D&C and final pathological diagnosis was approximately 76.2%, 16.8% of the tumor grade was upgraded and approximately 41.7% of the patients with these upgrades were upgraded from low-grade to high-grade, which was not different from the results of previous studies. However, inadequate grading may lead gynecologists to incorrectly assess the risk of LNM and select suboptimal treatment plans (6). In theory, radiomics can noninvasively obtain information about tumors and predict tumor heterogeneity and aggressiveness. Therefore, we compared the radiomics nomogram with the curettage results, and DCA reported that the radiomics nomogram can get higher net benefit. In addition, the NRI showed that the discrimination ability of the radiomics nomogram was significantly improved compared with that of D&C in the training and validation sets. Considering that the NRI measures the improvement of a certain threshold and cannot evaluate the overall improvement of the model, we recalculated the IDI. The IDI indicated that about five to 11 patients would benefit from the prediction of radiomics nomogram. In general, we believe that the radiomics nomogram has advantages over preoperative D&C in differentiating low-grade EC from high-grade EC.

With the rapid development of radiomics technology, a more precise and accurate quantitative assessment of lesions and radiomics has the advantages of being noninvasive and reproducible. We believe that radiomics will become a safer and more reliable clinical tool for predicting tumor grade and evaluating EC prognosis in the future.

Our study had some limitations. First, this retrospective study only included patients who met the inclusion and exclusion criteria, which might have resulted in selection bias. Second, all enrolled patients underwent diagnostic curettage before the MRI scan, which may cause the tumor volume seen on MRI to be smaller than the actual size, and the evaluation of tumor grade by the maximum diameter of the tumor in this study will be disturbed. Third, different field strengths and machine types may cause image heterogeneity. Therefore, we resampled and normalized the images and standardized the extracted features to reduce differences. Finally, this was a single-center small sample study, it cannot be denied that there may be an imbalance in the distribution of pathological features in the validation set. Therefore, a larger sample size and external validation are needed to verify the robustness and reproducibility of the radiomics nomogram.

In conclusion, we developed a radiomics nomogram based on MRI radiomics and clinical data that has good diagnostic performance for identifying high- and low-grade EC. The nomogram had a good net clinical benefit compared with that of D&C and provided an effective noninvasive tool for gynecologists to assess EC risk stratification before surgery.

## Data availability statement

The original contributions presented in the study are included in the article/**Supplementary Material**. Further inquiries can be directed to the corresponding author.

## Ethics statement

The studies involving human participants were reviewed and approved by The First Affiliated Hospital of Shihezi University School of Medicine. Written informed consent for participation was not required for this study in accordance with the national legislation and the institutional requirements.

## Author contributions

XY: Conceptualization, Data curation, Methodology, Writing–original draft. XH: Data curation, Software, Methodology, Formal analysis. SH: Data curation, Investigation. JW: Data curation, Investigation. Formal analysis. WF: Software, Supervision. HZ: Data curation. CW: Conceptualization, Supervision, Writing-review &editing. All authors contributed to the article and approved the submitted version.

## Supplementary material

Supplementary Figure 1
**(A)** Radiomics features were selected by least absolute shrinkage and selection operator (LASSO) logistic regression model in the training set. **(B)** The penalty parameter log (λ) was selected using 10-fold cross-validation through the minimum criterion, with the dashed line on the left representing the minimum log (λ) and the dashed line on the right representing log (λ) one standard error from the minimum.

Supplementary Figure 2 The clinical decision curves of three models for predicting high-grade endometrial cancer in the training **(A)** and validation sets **(B)**.

## References

[1] Koskas M Amant F Mirza M Creutzberg C. Cancer of the corpus uteri: 2021 update. Int J Gynecol Obstet: Off Organ Int Fed Gynaecology Obstetrics (2021); 155 (Suppl. S1): 45–60. doi: 10.1002/ijgo.13866 PMC9297903 34669196

[2] Fitzmaurice C Akinyemiju TF Al Lami FH Alam T Alizadeh-Navaei R Allen C. Global, regional, and national cancer incidence, mortality, years of life lost, years lived with disability, and disability-adjusted life-years for 29 cancer groups, 1990 to 2016: A systematic analysis for the global burden of disease study. JAMA Oncol (2018) 4(11):1553–68. doi: 10.1001/jamaoncol.2018.2706 PMC6248091 29860482

[3] Concin N Matias-Guiu X Vergote I Cibula D Mirza MR Marnitz S. ESGO/ESTRO/ESP guidelines for the management of patients with endometrial carcinoma. Int J Gynecol Cancer Off J Int Gynecological Cancer Soc (2021) 31(1):12–39. doi: 10.1136/ijgc-2020-002230 33397713

[4] Siegel RL Miller KD Jemal A. Cancer statistics, 2019. CA: Cancer J Clin (2019) 69(1):7–34. doi: 10.3322/caac.21551 30620402

[5] Bogani G Dowdy S Cliby W Ghezzi F Rossetti D Frigerio L. Management of endometrial cancer: issues and controversies. Eur J Gynaecological Oncol (2016) 37(1):6–12. 27048101

[6] Lukanović D Matjašič M Kobal B. Accuracy of preoperative sampling diagnosis for predicting final pathology in patients with endometrial carcinoma: A review. Trans Cancer Res (2020) 9(12):7785–96. doi: 10.21037/tcr-20-2228 PMC8798103 35117381

[7] Koh W Abu-Rustum N Bean S Bradley K Campos S Cho K. Uterine neoplasms, version 1.2018, NCCN clinical practice guidelines in oncology. J Natl Compr Cancer Netw (2018) 16(2):170–99. doi: 10.6004/jnccn.2018.0006 29439178

[8] Colombo N Creutzberg C Amant F Bosse T González-Martín A Ledermann J. ESMO-ESGO-ESTRO consensus conference on endometrial cancer: diagnosis, treatment and follow-up. Ann Oncol Off J Eur Soc Med Oncol (2016) 27(1):16–41. doi: 10.1093/annonc/mdv484 26634381

[9] Bhardwaj V Sharma A Parambath S Gul I Zhang X Lobie P. Machine learning for endometrial cancer prediction and prognostication. Front Oncol (2022) 12:852746. doi: 10.3389/fonc.2022.852746 35965548 PMC9365068

[10] Reyes-Pérez JA Villaseñor-Navarro Y Jiménez de Los Santos ME Pacheco-Bravo I Calle-Loja M Sollozo-Dupont I. The apparent diffusion coefficient (ADC) on 3-T MRI differentiates myometrial invasion depth and histological grade in patients with endometrial cancer. Acta Radiologica (Stockholm Sweden 1987) (2020) 61(9):1277–86. doi: 10.1177/0284185119898658 31955608

[11] Kakkar C Gupta K Jain K Narang V Singh A Saggar K. Diagnostic accuracy of calculated tumor volumes and apparent diffusion coefficient values in predicting endometrial cancer grade. Int J Appl Basic Med Res (2022) 12(1):37–42. doi: 10.4103/ijabmr.ijabmr_553_21 35265479 PMC8848552

[12] Kumar V Gu Y Basu S Berglund A Eschrich SA Schabath MB. Radiomics: the process and the challenges. Magnetic resonance Imaging (2012) 30(9):1234–48. doi: 10.1016/j.mri.2012.06.010 PMC3563280 22898692

[13] Kickingereder P Burth S Wick A Götz M Eidel O Schlemmer HP. Radiomic profiling of glioblastoma: Identifying an imaging predictor of patient survival with improved performance over established clinical and radiologic risk models. Radiology (2016) 280(3):880–9. doi: 10.1148/radiol.2016160845 27326665

[14] Lee SH Park H Ko ES. Radiomics in breast imaging from techniques to clinical applications: A review. Korean J Radiol (2020) 21(7):779–92. doi: 10.3348/kjr.2019.0855 PMC7289696 32524780

[15] Rodríguez-Ortega A Alegre A Lago V Carot-Sierra JM Ten-Esteve A Montoliu G. Machine learning-based integration of prognostic magnetic resonance imaging biomarkers for myometrial invasion stratification in endometrial cancer. J Magnetic Resonance Imag JMRI (2021) 54(3):987–95. doi: 10.1002/jmri.27625 33793008

[16] Long L Sun J Jiang L Hu Y Li L Tan Y. MRI-Based traditional radiomics and computer-vision nomogram for predicting lymphovascular space invasion in endometrial carcinoma. Diagn Interventional Imag (2021) 102(7-8):455–62. doi: 10.1016/j.diii.2021.02.008 33741266

[17] Liu XF Yan BC Li Y Ma FH Qiang JW. Radiomics nomogram in assisting lymphadenectomy decisions by predicting lymph node metastasis in early-stage endometrial cancer. Front Oncol (2022) 12:894918. doi: 10.3389/fonc.2022.894918 35712484 PMC9192943

[18] Amant F Mirza MR Koskas M Creutzberg CL. Cancer of the corpus uteri. Int J Gynaecol Obstet (2018) 143 Suppl 2:37–50. doi: 10.1002/ijgo.12612 30306580

[19] Jose T Singh A Vardhan S. Pre-surgical staging in endometrial cancer: An opportunity for risk stratification and triage? Med J Armed Forces India (2021) 77(2):205–13. doi: 10.1016/j.mjafi.2020.09.009 PMC8042510 33867639

[20] Zamani N Modares Gilani M Zamani F Zamani MH. Utility of pelvic MRI and tumor markers HE4 and CA125 to predict depth of myometrial invasion and cervical involvement in endometrial cancer. J Family Reprod Health (2015) 9(4):177–83. PMC4818380 27047564

[21] Teng F Zhang YF Wang YM Yu J Lang X Tian WY. Contrast-enhanced MRI in preoperative assessment of myometrial and cervical invasion, and lymph node metastasis: Diagnostic value and error analysis in endometrial carcinoma. Acta Obstet Gynecol Scand (2015) 94(3):266–73. doi: 10.1111/aogs.12570 25545203

[22] Wang Y Bi Q Deng Y Yang Z Song Y Wu Y. Development and validation of an MRI-based radiomics nomogram for assessing deep myometrial invasion in early stage endometrial adenocarcinoma. Acad Radiol (2022), S1076-6332(22)00320–8. doi: 10.1016/j.acra.2022.05.017 35778306

[23] Yan BC Li Y Ma FH Feng F Sun MH Lin GW. Preoperative assessment for high-risk endometrial cancer by developing an MRI- and clinical-based radiomics nomogram: A multicenter study. J Magnetic Resonance Imag JMRI (2020) 52(6):1872–82. doi: 10.1002/jmri.27289 32681608

[24] Kim SI Kim JW. Endometrial cancer. N Engl J Med (2021) 384(6):586. doi: 10.1056/NEJMc2035378 33567203

[25] Degez M Caillon H Chauviré-Drouard A Leroy M Lair D Winer N. Endometrial cancer: A systematic review of HE4, REM and REM-b. Clinica Chimica Acta; Int J Clin Chem (2021) 515:27–36. doi: 10.1016/j.cca.2020.12.029 33388311

[26] Zheng T Yang L Du J Dong Y Wu S Shi Q. Combination analysis of a radiomics-based predictive model with clinical indicators for the preoperative assessment of histological grade in endometrial carcinoma. Front Oncol (2021) 11:582495. doi: 10.3389/fonc.2021.582495 34235069 PMC8255911

[27] Panyavaranant P Manchana T. Preoperative markers for the prediction of high-risk features in endometrial cancer. World J Clin Oncol (2020) 11(6):378–88. doi: 10.5306/wjco.v11.i6.378 PMC7450819 32874951

[28] Sala E Mema E Himoto Y Veeraraghavan H Brenton JD Snyder A. Unravelling tumour heterogeneity using next-generation imaging: radiomics, radiogenomics, and habitat imaging. Clin Radiol (2017) 72(1):3–10. doi: 10.1016/j.crad.2016.09.013 27742105 PMC5503113

[29] Aerts HJ Velazquez ER Leijenaar RT Parmar C Grossmann P Carvalho S. Decoding tumour phenotype by noninvasive imaging using a quantitative radiomics approach. Nat Commun (2014) 5:4006. doi: 10.1038/ncomms5006 24892406 PMC4059926

[30] Bereby-Kahane M Dautry R Matzner-Lober E Cornelis F Sebbag-Sfez D Place V. Prediction of tumor grade and lymphovascular space invasion in endometrial adenocarcinoma with MR imaging-based radiomic analysis. Diagn Interventional Imag (2020) 101(6):401–11. doi: 10.1016/j.diii.2020.01.003 32037289

[31] Visser N Reijnen C Massuger L Nagtegaal I Bulten J Pijnenborg J. Accuracy of endometrial sampling in endometrial carcinoma: A systematic review and meta-analysis. Obstetrics Gynecol (2017) 130(4):803–13. doi: 10.1097/aog.0000000000002261 28885397

