# Supplementary figures and images for: Multiparametric magnetic resonance imaging-based radiomics nomogram for predicting tumor grade in endometrial cancer
Source: Front Oncol. 2023 Feb 21;13:1081134. doi: 10.3389/fonc.2023.1081134 (PMC9989162; doi:10.3389/fonc.2023.1081134)

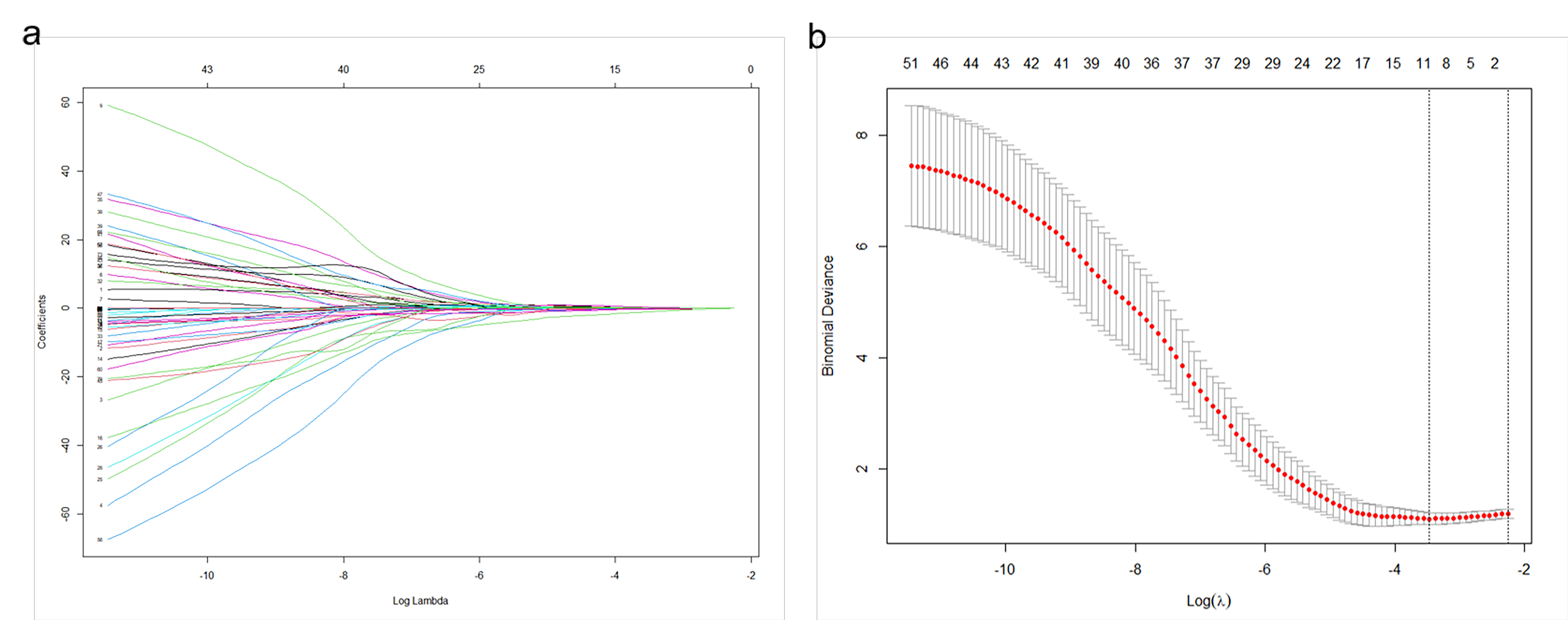

Supplement: Supplementary Figure 1 — (A) Radiomics features were selected by least absolute shrinkage and selection operator (LASSO) logistic regression model in the training set. (B) The penalty parameter log (λ) was selected using 10-fold cross-validation through the minimum criterion, with the dashed line on the left representing the minimum log (λ) and the dashed line on the right representing log (λ) one standard error from the minimum. [file Image_1.tif]

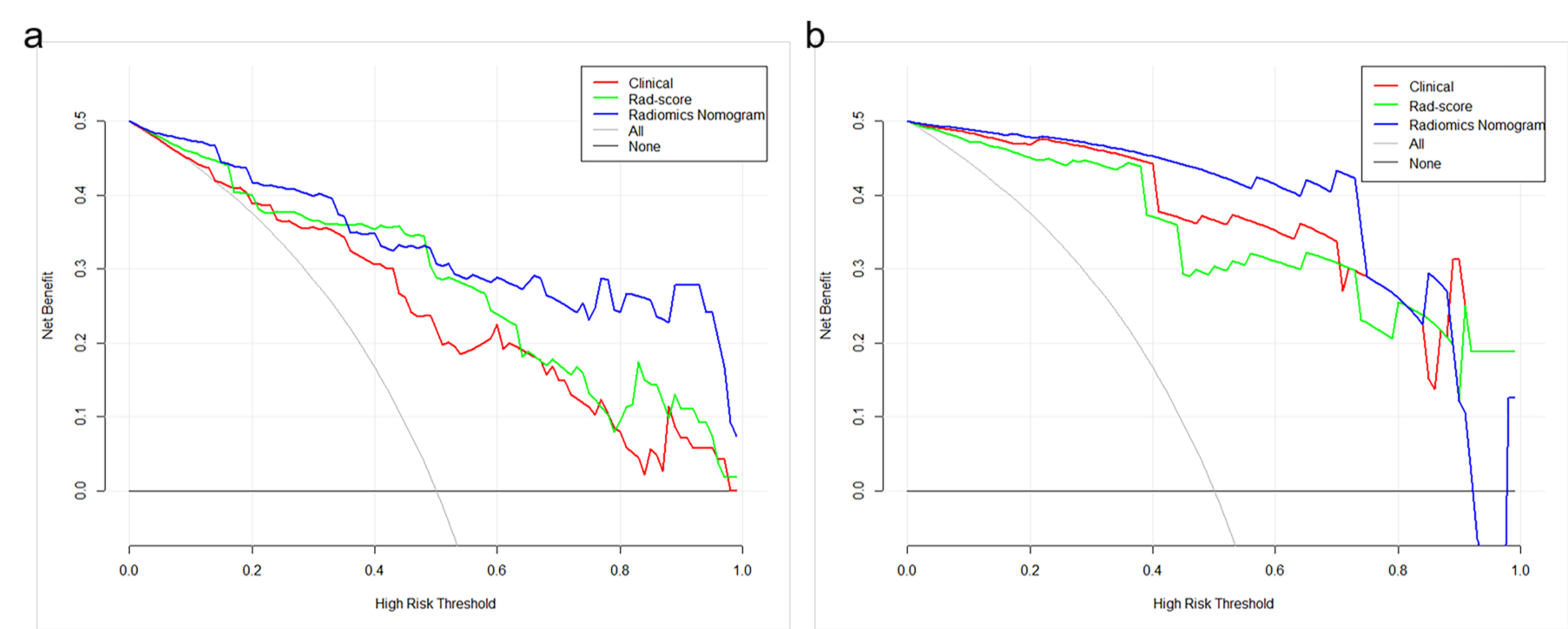

Supplement: Supplementary Figure 2 — The clinical decision curves of three models for predicting high-grade endometrial cancer in the training (A) and validation sets (B). [file Image_2.tif]
