# Supplementary material for: Multiparametric magnetic resonance imaging-based radiomics nomogram for predicting tumor grade in endometrial cancer
Source: Front Oncol. 2023 Feb 21;13:1081134. doi: 10.3389/fonc.2023.1081134 (PMC9989162; doi:10.3389/fonc.2023.1081134)
Supplement: Supplementary file 3 [file Table_1.docx]

Supplementary Table S1A: Detailed MRI examination's parameters.

| parameters | GE Discovery MR750W 3.0T | | | | | Philips Multiva 1.5T | | | | |
| --- | --- | --- | --- | --- | --- | --- | --- | --- | --- | --- |
|  | T1WI (FSE) | T2WI (FSE) | T2WI (FS FSE) | DWI | DCE | T1WI (FSE) | T2WI (FSE) | T2WI (FS FSE) | DWI | DCE |
| Scan Plane | Axial | Sagittal/coronal | Axial | Axial | Axial | Axial | Sagittal/coronal | Axial | Axial | Axial |
| FOV (mm) | 280 | 240 | 280 | 320 | 380 | 220 | 220 | 220 | 220 | 240 |
| Matrix | 320$\times$224 | 320$\times$256 | 320$\times$224 | 128$\times$128 | 256$\times$256 | 256$\times$256 | 256$\times$256 | 256$\times$256 | 256$\times$256 | 320$\times$320 |
| TR/TE (ms) | 500/8.3 | 5876/85 | 4720/90 | 3000/70 | 3.2/1.5 | 520/10 | 4200/110 | 4000/80 | 6000/64 | 4.5/1.4 |
| Thickness/Gap (mm) | 5/1 | 5/1 | 5/1 | 5/1 | 3.5/0 | 4/0.5 | 5/1 | 4/0.5 | 4/0.4 | 3/0 |
| slices | 24 | 24 | 24 | 24 | 88 | 21 | 21 | 21 | 21 | 55 |
| NEX | 1 | 2 | 4 | 6 | 0.69 | 1 | 2 | 2 | 2 | 1 |
| Scan time | 2min 13s | 2min 32s/2min 35s | 2min 41s | 1min 20s | 3min 25s | 2min17s | 3min 1s/3min 1s | 2min 24s | 3min 18s | 3min |
| b values (s/mm^2^) | NA | NA | ­ NA | 0, 800 | NA | NA | NA | NA | 0, 800 | NA |

T2WI, T2-weighted imaging; DWI, diffusion weighted imaging; DCE, dynamic contrast-enhanced; TR, repetition time; TE, echo time; FOV, field of view; NEX, number of excitation.

Supplemental Table S2A: Detailed information on the radiomics features.

| Feature classes | Numbers | Definite features |
| --- | --- | --- |
| Shape | 14 features | Elongation, Flatness, Least Axis Length, Major Axis Length, Maximum 2D Diameter (Column), Maximum 2D Diameter (Row), Maximum2D Diameter (Slice), Maximum 3D Diameter, Mesh Volume, Minor Axis Length, Sphericity, Surface Area, Surface Volume Ratio, Voxel Volume, |
| First Order | 18 features | 10 Percentile, 90Percentile, Energy, Entropy, Interquartile Range, Kurtosis, Maximum, Mean Absolute Deviation, Mean, Median, Minimum, Range, Robust Mean Absolute Deviation, Root Mean Squared, Skewness, Total Energy, Uniformity, Variance |
| Gray Level Co-occurrence Matrix (GLCM) | 24 features | Autocorrelation, Cluster Prominence, Cluster Shade, Cluster Tendency, Contrast, Correlation, Difference Average, Difference Entropy, Difference Variance, Id, Idm, Idmn, Idn, Imc1, Imc2, Inverse Variance, Joint Average, Joint Energy, Joint Entropy, MCC, Maximum Probability, Sum Average, Sum Entropy, Sum Squares |
| Gray Level Dependence Matrix (GLDM) | 14 features | Dependence Entropy, Dependence Non-Uniformity, Dependence Non-Uniformity Normalized, Dependence Variance, Gray Level Non-Uniformity, GrayLevelVariance, High Gray Level Emphasis, Large Dependence Emphasis, Large Dependence High Gray Level Emphasis, Large Dependence Low Gray Level Emphasis, Low Gray Level Emphasis, Small Dependence Emphasis, Small Dependence High Gray Level Emphasis, Small Dependence Low Gray Level Emphasis |
| Gray Level Size Zone Matrix (GLSZM) | 16 features | Gray Level Non-Uniformity, Gray Level Non-Uniformity Normalized, GrayLevelVariance, High Gray Level Zone Emphasis, Large Area Emphasis, Large Area High Gray Level Emphasis, Large Area Low Gray Level Emphasis, Low Gray Level Zone Emphasis, Size Zone Non-Uniformity, Size Zone Non-Uniformity Normalized, Small Area Emphasis, Small Area High Gray Level Emphasis, Small Area Low Gray Level Emphasis, Zone Entropy, Zone Percentage, Zone Variance |
| Gray Level Run Length Matrix (GLRLM) | 16 features | Gray Level Non-Uniformity, Gray Level Non-Uniformity Normalized, GrayLevelVariance, High Gray Level Run Emphasis, Long Run Emphasis, Long Run High Gray Level Emphasis, Long Run Low Gray Level Emphasis, Low Gray Level Run Emphasis, Run Entropy, Run Length Non-Uniformity, Run Length Non-Uniformity Normalized, Run Percentage, Run Variance, Short Run Emphasis, Short Run High Gray Level Emphasis, Short Run Low Gray Level Emphasis |
| Neighbouring Gray Tone Difference Matrix (NGTDM) | 5 features | Busyness, Coarseness, Complexity, Contrast, Strength |

Supplemental Tabel S3A: Characteristics of 143 patients with endometrial cancer.

| Variable | High-grade (n$=$35) | Low-grade (n$=$108) | *P*-value |
| --- | --- | --- | --- |
| Age (years) | 60.97$\pm$11.32 | 53.76$\pm$9.57 | 0.001 |
| CA125 | 54.10$\pm$49.79 | 37.22$\pm$51.50 | 0.091 |
| HE4 | 148.67$\pm$135.12 | 94.91$\pm$70.71 | 0.001 |
| Max tumor diameter | 64.21$\pm$38.89 | 47.20$\pm$18.08 | 0.062 |
| MR_DMI | 23 (65.71%) | 26 (24.07%) | $<$0.001 |
| MR_CSI | 15 (42.86%) | 14 (12.96%) | $<$0.001 |
| MR_LNM | 12 (34.28%) | 4 (3.70%) | $<$0.001 |

CA125, cancer antigen 125; HE4, human epididymis protein 4; Max, maximum; MR_DMI, MRI-reported deep myometrium invasion; MR_CSI, MRI-reported cervical stromal invasion; MR_LNM, MRI-reported lymph node metastasis.
